# The synthesis of the novel *Escherichia coli* toxin—colibactin and its mechanisms of tumorigenesis of colorectal cancer

**DOI:** 10.3389/fmicb.2024.1501973

**Published:** 2024-12-18

**Authors:** Guojing Zhang, Daqing Sun

**Affiliations:** Pediatric Surgery Department, Tianjin Medical University General Hospital, Tianjin, China

**Keywords:** colibactin, *Escherichia coli*, PKS gene, mechanism, synthesis

## Abstract

*Escherichia coli* is part of the normal flora of the human gut and performs vital functions; however, certain strains can cause disease in the host, impairing gut function and adversely affecting overall health. The pks gene cluster in the *E. coli* B2 serogroup encodes colibactin, a secondary metabolite and a potential gut toxin. However, the mechanism underlying colibactin production in *E. coli* is complex, and the function of the pks gene cluster is not fully understood. This review explores the complex mechanisms and processes by which the pks island in *E. coli* produces colibactin, clarifying the specific role played by the *clbA-S* genes within it. It also reveals the toxic effects of colibactin on the host cell’s DNA and elaborates the mechanisms that may be important in inducing the development of colorectal cancer, such as single-base substitution (SBS), small insertion/deletion (small indel) features (ID-pks), inter-chromosomal linkages (ICLs), and DNA double-strand breaks (DSBs). The elucidation of these mechanisms is of great significance for the further exploration and development of related drugs.

## 1 Introduction

*Escherichia coli*, which is part of the normal flora of the human gut, plays a significant yet complex role in regulating intestinal function (Crosby et al., 2019). However, certain strains of *E. coli* can cause disease in the host, impairing gut function and adversely affecting overall health (Kaper et al., 2004; Vila et al., 2016). The *E. coli* B2 serogroup harbors the pks gene cluster, which encodes colibactin, a molecule potentially involved in the pathogenicity of the disease-causing strains in the gut (Abram et al., 2021; Gatya Al-Mayahie et al., 2022). However, the mechanism underlying its production in *E. coli* is complex, and the function of the pks gene cluster remains to be fully elucidated. Recent findings suggest that colibactin can induce DNA damage and genomic instability in human intestinal cells, leading to cellular aging and increased intestinal permeability, and may be important in the development of colorectal cancer (CRC) (Dougherty and Jobin, 2021; Dubinsky et al., 2020; Miyasaka et al., 2024; Tang-Fichaux et al., 2021). Nevertheless, as a secondary metabolite of *E. coli*, colibactin is structurally unstable and difficult to isolate, presenting a significant challenge to investigating the mechanisms underlying its actions. Previous reviews on PKS Island have largely focused on the structure, regulatory mechanisms, and tumorigenic mechanisms of colibactin (Chagneau et al., 2022). However, these articles lack in-depth descriptions of the production of colibactin and its mechanisms of inducing colorectal cancer. This article delves into the origin of colibactin, its biosynthetic pathway, and the mechanisms by which it induces colorectal tumors, with a particular focus on unraveling the genetic aspects of colibactin-induced tumorigenesis, thereby providing a foundation for further investigation into the effects of colibactin on human diseases.

## 2 Escherichia coli

*E. coli* belongs to the family Enterobacteriaceae within the class *γ*-Proteobacteria. It primarily colonizes the intestines of humans and animals and inhibits the colonization of other bacteria (Crosby et al., 2019). Although it plays a vital role in the intestine, *E. coli* has also been isolated from patients with bloodstream and urinary tract infections. The actions of *E. coli* are multifaceted, colonizing the gastrointestinal tract as a commensal bacterium, while also acting as a pathogen to elicit a spectrum of symptoms in organs, such as the gastrointestinal and urinary systems (Kaper et al., 2004; Vila et al., 2016).

*E. coli* can be classified into distinct phylogenetic groups, namely A, B1, B2, D, E, F, and the closely related groups C and G based on its genetic characteristics and developmental traits (Abram et al., 2021). Group B2 is noted for the increased adherence and invasion of its strains into intestinal cells, potentially contributing to bacterial colonization in CRC tumor tissue. Strains of group B2 harbor virulence-associated genes, increasing their potential cytotoxicity, thereby leading to cell death and inflammatory responses (Gatya Al-Mayahie et al., 2022; Nouri et al., 2016).

Depending on the site of colonization, *E. coli* strains are categorized into extraintestinal pathogenic Enterobacteriaceae (ExPEC) and intestinal pathogenic Enterobacteriaceae (InPEC). ExPEC strains primarily colonize extraintestinal organs, such as the peritoneum, vagina, cervix, urinary tract, and bloodstream, causing infection at these sites (Jang et al., 2017; Sharma et al., 2016; Vogeleer et al., 2014). Conversely, InPEC strains primarily inhabit the gastrointestinal tract and are associated with gastrointestinal diseases, including diarrhea, constipation, inflammatory bowel disease, and irritable bowel syndrome. These strains produce toxins and are classified based on their pathogenic characteristics and mechanisms (Alizade et al., 2019; Jang et al., 2017). InPEC group strains frequently exhibit a regional distribution pattern, and their contamination of food and water frequently leads to outbreaks of related diseases in developing countries, but they rarely cause extraintestinal infections and are less likely to colonize a healthy host (Alizade et al., 2019; Farrokh et al., 2013; Flores-Mireles et al., 2015).

The most prominent subgroup within the InPEC group is colibactin-producing *E. coli* (pEPEC) (Oliero et al., 2022; Taoum et al., 2022). Recent evidence suggests that the colonization of pEPEC in the gastrointestinal tract may be a significant contributing factor to the development of gastrointestinal tumors. Moreover, *E. coli* strains isolated from patients with colonic tumors exhibit a significantly higher potential of colicin production (Tariq et al., 2022; Yoshikawa et al., 2020; Zhou S. et al., 2021; Zhou T. et al., 2021).

## 3 The pks island in *Escherichia coli*

The hybrid non-ribosomal peptide synthetase-polyketide synthetase (NRPS-PKS) biosynthetic gene cluster, known as the pks or clb island, comprises 19 genes (clb A–S), which encode three PKSs, three NRPSs, two NRPS/PKS hybrids, MATE transporters, resistance genes, and nine additional trimming and accessory enzymes (Tang J. W. et al., 2022; Tang S. et al., 2022). These enzymes participate in the biosynthetic pathway of colibactin, a peptidoglycan-genotoxin polyketide (Kalantari et al., 2023; Wong et al., 2022; Xia et al., 2020). Colibactin is produced by specific strains of *E. coli* and related bacteria, such as *Klebsiella pneumoniae*, *Citrobacter koseri*, and *Enterobacter cloacae*, collectively known as pks + strains (colibactin-producing *E. coli*) (Kalantari et al., 2023; Tang J. W. et al., 2022; Tang S. et al., 2022). The pks island, containing virulence-associated genes, features a unique genetic locus and is often linked to mobile genetic elements. It is approximately 54–56 kb in size and may vary among different pks-positive strains. Moreover, the number of base pairs is not uniform. In bacteria, it can exist as a mobile element, such as transposons, plasmids, and other elements, facilitating transfer between different bacterial species. Alternatively, it can integrate into the genome of the bacterial species. It encodes a mixture of colibactin and other metabolites and intermediates, influencing host-pathogen interactions (Vizcaino et al., 2014).

The pks island is predominantly found in *E. coli* strains of the B2 group but is also present in strains from groups A and B1, each with distinct genetic structures, indicating multiple introduction mechanisms into *E. coli*. Recent studies indicated a higher prevalence of pks + *E. coli* in CRC patients than in healthy individuals. Nouri et al. (2021) identified pks in 23% of CRC patients compared to 7.1% in the control group, corroborating prior findings by Buc et al. (2013) who detected the gene in 39.5% of CRC patients and 12.9% of diverticulosis patients. Thus, the pks island is a potential biomarker for CRC development. Moreover, it is highly conserved across various Enterobacteriaceae species and performs diverse functions beyond its potential role in CRC development (Chagneau et al., 2022).

## 4 Colibactin

Research on colibactin began in 2006 when Nougayrede infected eukaryotic cells with *E. coli* B2, which blocked mitosis and induced megakaryocyte proliferation, characteristics associated with the widespread pks island. This island encodes numerous non-ribosomal peptide and polyketide synthetases, causing DNA double-strand breaks (DSBs) and activating DNA damage checkpoint pathways, which lead to cell cycle arrest and cell death (Nougayrède et al., 2006). As already mentioned, colibactin is linked to human health (Silpe et al., 2022). However, despite its significant biological activities, it has not been isolated and structurally characterized by traditional methods, and thus information on its chemical structure mainly comes from bioinformatics and biochemical analyses.

### 4.1 Synthesis, export, and self-resistance of colibactin

Colibactin biosynthesis is a multi-stage process requiring specific enzymes. The pks island encodes the proteins constituting the biosynthesis pathway, which is divided into five steps (Chagneau et al., 2022).

- *Step 1*: Activation of clb genes induces the expression of precursor proteins (ClbA, ClbB, ClbC, and ClbD) involved in colibactin biosynthesis (Hirayama et al., 2022). The gene *clbR* encodes a transcriptional activator that self-regulates and activates *clbB* transcription, with longer VNTR sequences enhancing *clbR* expression.
- *Step 2*: PKS, NRPS, and NRPS-PKS metabolite synthesis. Here, ClbD, ClbE, ClbF, and ClbG encode AM-ACP; ClbC, ClbL, and ClbO encode PKS; ClbH, ClbJ, and ClbN encode NRPS; and ClbB and ClbK are responsible for the binding of PKS to NRPS to form the PKS-NRPS complex to facilitate metabolite synthesis. NRPS and PKS use amino acids, coenzyme A, and aminoacyl-glycerol-phosphate to extend the metabolite length. The PPTase ClbA activates NRPS and PKS by adding a phosphoprotein arm that carries NRP-PK metabolites (Chagneau et al., 2022). The ClbA enzyme catalyzes the cyclization of tryptophan into a cyclodiene, a key step in the genetic toxicity of colibactin (Tang-Fichaux et al., 2020). The ClbC enzyme catalyzes the condensation of aspartate to precolibactin, producing a new intermediate structure, and the thioesterase ClbQ modifies the metabolite.
- *Step 3*: Formation of precolibactin. The NRPS-PKS complex synthesizes metabolites, which are dimerized into precolibactin, the precursor of colibactin, by ClbL.
- *Step 4*: Translocation of precolibactin. Following its formation, precolibactin is transported to the periplasm through the MATE transporter encoded by *clbM*.
- *Step 5*: Release of colibactin. Following step 4, the ClbP enzyme cleaves specific bonds within the intermediate structure, forming a cyclic ether colibactin molecule, which is then released (Chagneau et al., 2022; Velilla et al., 2023). The *clbP* gene encodes a peptidase, ClbP, which is structurally and functionally homologous to post-translationally modified peptidases (Luo et al., 2011). Colibactin is exported from bacterial cells during this step, a crucial process for its genetic toxicity (Clay et al., 2022).

The mechanisms by which colibactin is exported from bacteria, enters host cells, and is transported to the nucleus are not yet understood. The double-headed DNA-binding domain of colibactin binds to both strands of the host genome DNA, inducing covalent interstrand crosslinks. Upon re-entry into the producing bacteria, the colibactin moiety is hydrolyzed by the self-resistance protein ClbS, which also binds to and protects bacterial DNA. The bacterial DNA repair system provides an additional self-protective layer, with ClbS possibly assisting with repair (Chagneau et al., 2022).

### 4.2 Regulation of pks-related gene expression

The expression of several pks genes is regulated by oxygen, iron, and carbon availability. Hypoxic conditions induce increased expression of ClbB (Chen et al., 2022). Transcription activation of the pks island by clbR in response to iron availability is mediated by iron depletion, which enhances clbR transcription, thereby increasing colibactin production (Wallenstein et al., 2020). Iron also activates *clbA* transcription, regulating its expression and participating in the synthesis of iron carriers (Martin et al., 2013; Tronnet et al., 2016). Iron supplementation inhibits clbA and clbR expression, suggesting that colibactin and iron carrier regulation both respond to iron availability. RyhB inhibits clbA expression and colibactin production, while promoting iron carrier expression. ClbA not only plays a significant role in the activation of colibactin biosynthesis but also participates in the activation of the biosynthetic pathways of iron carriers, such as yersiniabactin, salmochelin, and enterobactin, through certain biological processes.

**Figure 1:**
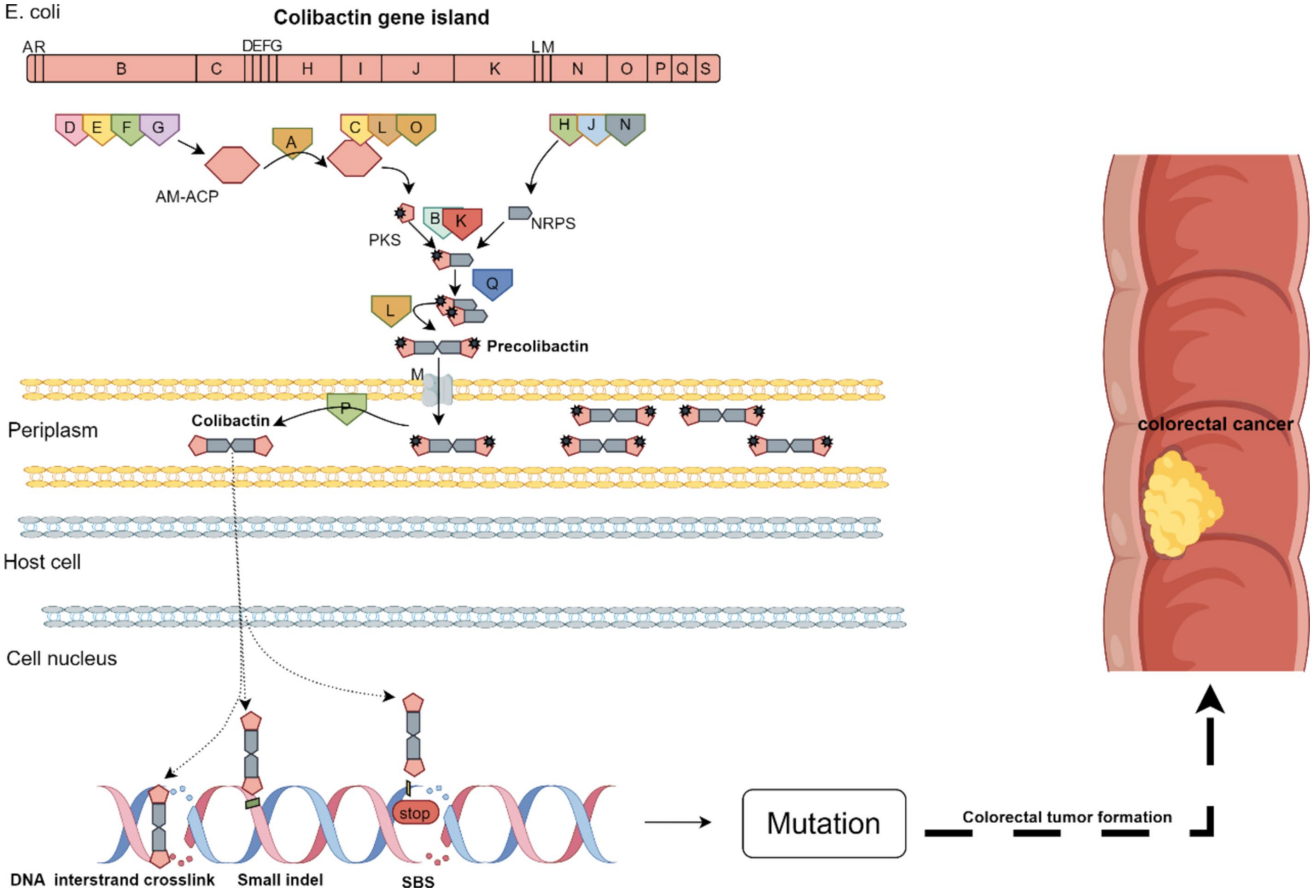
The synthesis of colibactin and its mechanisms of inducing colorectal cancer.

Additionally, the expression of pks genes is significantly correlated with the presence of carbon sources, with some carbon sources, such as acetic acid and glucose, leading to a significant increase in the expression of pks genes (Homburg et al., 2007). The key translational regulator of the carbon-storage regulator system, CsrA, can exert inhibitory effects on the synthesis of colibactin by suppressing the expression of some pks genes, such as *clbR* and *clbQ* (Rehm et al., 2021; Wong et al., 2022) (Figure 1).

Further, the regulation of pks gene expression is linked to the synthesis of spermidine and polyphosphates. Spermidine is closely related to the synthesis of SpeE, which can promote the expression of the *clb* gene, one of the essential factors for the synthesis of colibactin (Chagneau et al., 2019). The enzyme PPK, which catalyzes inositol hexakisphosphate synthesis, is crucial for *clb* gene expression and colibactin production (Tang-Fichaux et al., 2020). Inositol hexakisphosphate, which plays a role in bacterial responses to stressors, such as oxidants, nutrient depletion, and heavy metals, is frequently overexpressed. ClbP mutations can prevent deacetylation, promoting the accumulation of stable precolibactin. Boronic acid inhibitors, mimicking the colibactin precursor adhesin, effectively inhibit the ClbP peptidase, enabling precise regulation of colibactin production and serving as a valuable tool for studying the biological functions of colibactin (Volpe et al., 2023).

### 4.3 Colibactin structure

Colibactin is an unstable secondary metabolite that cannot be isolated directly from the producing *E. coli*. The structure and mechanism of action of colibactin have been investigated through biosynthetic and genetic, enzymology, large-scale fermentation and separation, chemical synthesis, and DNA alkylation methods (Wernke et al., 2020). Studies indicate that colibactin is a heterodimer with two DNA-reactive cyclopropane residues, featuring a structure of two thiazole rings linked by two carbon connectors, and likely exists as *α*-amino ketones post-biosynthesis (Zhou S. et al., 2021; Zhou T. et al., 2021). However, current synthetic studies have confirmed that this α-amino ketone is unstable under aerobic oxidation; under mild conditions, the resulting oxidation products do not induce nucleophilic cleavage. These findings offer a molecular-level explanation for the instability of colibactin and may help elucidate the necessity of cell-to-cell contact for the genetic toxic effects.

## 5 Mechanism of colibactin

The instability, low yield, and complex biosynthetic process of colibactin have posed significant challenges for structural elucidation (Bian et al., 2013; Brotherton and Balskus, 2013). *In vitro* infection with *E. coli* harboring the clb island induces DSBs in human cells, resulting in cell cycle arrest and subsequent cell death (Nougayrède et al., 2006). Physiological studies indicate that clb + bacteria cause DNA damage and genomic instability in intestinal cells *in vivo*, leading to cellular senescence (Cougnoux et al., 2014; Secher et al., 2013), increased intestinal permeability (Payros et al., 2014), and colorectal tumor formation in chronic intestinal inflammation mouse models (Cougnoux et al., 2014; Tomkovich et al., 2017). Notably, evidence suggests that pEPEC is associated with CRC (Dougherty and Jobin, 2021; Dubinsky et al., 2020; Miyasaka et al., 2024; Tang-Fichaux et al., 2021).

### 5.1 Toxic effects of colibactin on host cells (DSBs)

This genotoxin causes DNA damage by altering Wnt proteins and preventing *β*-catenin degradation, which results in reactive oxygen species production and DSBs. This further results in a brief cell cycle arrest at the G2-M phases, followed by cell death (Iyadorai et al., 2020). Recent studies indicate that colibactin induces DNA DSBs via copper-mediated oxidative cleavage. Colibactin binds to copper in the intestinal lumen, forming a colibactin-copper complex, which is then rapidly transported into epithelial cells. O_2_ production coordinated by the colibactin-copper complex induces the generation of ‘activated colibactin,’ which attacks and cleaves DNA (Li et al., 2019).

### 5.2 Single-base substitution (SBS), small insertion/deletion (small indel) features (ID-pks), and inter-chromosomal linkages (ICLs)

Microinjection of pks + *E. coli* into human organ lumens increases SBS levels, with a tendency for T > N substitution. Exposure to pks + *E. coli* induces a characteristic small indel feature (ID-pks), marked by a single T deletion on T homopolymers. Moreover, pks + *E. coli* induces ICLs and DSBs in epithelial cell lines and CRC mouse models, thereby promoting tumorigenesis. This damage induces mutations and genomic instability, promoting carcinogenesis and tumor development (Arthur et al., 2012; Buc et al., 2013; Dejea et al., 2018). This pks feature also exists in non-colorectal tumors, such as oral squamous cell carcinoma, which exhibits features very similar to SBS-pks and ID-pks (Pleguezuelos-Manzano et al., 2020).

### 5.3 Other mechanisms of colibactin

Colibactin can activate phage replication and release, allowing phages to infect other bacteria and exert their effects (Silpe et al., 2022). Evidence also indicates that pEPEC can enhance chemotherapy drug resistance by promoting epithelial-to-mesenchymal transition and the emergence of cancer stem cells (Dalmasso et al., 2024).

## 6 Discussion

Current research on colibactin has become relatively in-depth, with its role in inducing DSBs in DNA likely being an important mechanism for tumorigenesis, especially in CRC. Colibactin-related tumorigenic activities have also been detected in other tumors. Moreover, colibactin exhibits pathogenic effects in non-tumor diseases, but its elusive signaling pathways and a lack of full understanding of its mechanisms pose challenges. As an unstable secondary metabolite with low yield and a complex biosynthetic process, colibactin cannot be directly isolated from the producing *E. coli*, making structural elucidation and subsequent in-depth research on its mechanism of action highly challenging. Furthermore, the mechanisms by which *E. coli* produces colibactin, particularly the release and hydrolysis of colibactin, are not fully understood, highlighting the importance of further exploration of its biosynthetic mechanism for drug development.
